# The Preferred Substrates for Transglutaminase 2 in a Complex Wheat Gluten Digest Are Peptide Fragments Harboring Celiac Disease T-Cell Epitopes

**DOI:** 10.1371/journal.pone.0014056

**Published:** 2010-11-19

**Authors:** Siri Dørum, Magnus Ø. Arntzen, Shuo-Wang Qiao, Anders Holm, Christian J. Koehler, Bernd Thiede, Ludvig M. Sollid, Burkhard Fleckenstein

**Affiliations:** 1 Centre for Immune Regulation, Institute of Immunology, University of Oslo, Oslo, Norway; 2 The Biotechnology Centre of Oslo, University of Oslo, Oslo, Norway; 3 Proteomics Core Facility, Oslo University Hospital-Rikshospitalet, Oslo, Norway; 4 Proteomics Core Facility, Norwegian University of Life Sciences, Ås, Norway; 5 Centre for Immune Regulation, Institute of Immunology, Oslo University Hospital-Rikshospitalet, Oslo, Norway; Monash University, Australia

## Abstract

**Background:**

Celiac disease is a T-cell mediated chronic inflammatory disorder of the gut that is induced by dietary exposure to gluten proteins. CD4+ T cells of the intestinal lesion recognize gluten peptides in the context of HLA-DQ2.5 or HLA-DQ8 and the gluten derived peptides become better T-cell antigens after deamidation catalyzed by the enzyme transglutaminase 2 (TG2). In this study we aimed to identify the preferred peptide substrates of TG2 in a heterogeneous proteolytic digest of whole wheat gluten.

**Methods:**

A method was established to enrich for preferred TG2 substrates in a complex gluten peptide mixture by tagging with 5-biotinamido-pentylamine. Tagged peptides were isolated and then identified by nano-liquid chromatography online-coupled to tandem mass spectrometry, database searching and final manual data validation.

**Results:**

We identified 31 different peptides as preferred substrates of TG2. Strikingly, the majority of these peptides were harboring known gluten T-cell epitopes. Five TG2 peptide substrates that were predicted to bind to HLA-DQ2.5 did not contain previously characterized sequences of T-cell epitopes. Two of these peptides elicited T-cell responses when tested for recognition by intestinal T-cell lines of celiac disease patients, and thus they contain novel candidate T-cell epitopes. We also found that the intact 9mer core sequences of the respective epitopes were not present in all peptide substrates. Interestingly, those epitopes that were represented by intact forms were frequently recognized by T cells in celiac disease patients, whereas those that were present in truncated versions were infrequently recognized.

**Conclusion:**

TG2 as well as gastrointestinal proteolysis play important roles in the selection of gluten T-cell epitopes in celiac disease.

## Introduction

Celiac disease is an intestinal chronic inflammatory disorder elicited by dietary wheat gluten and related proteins from barley and rye. The disease is characterized by malabsorption, flattening of mucosa and the presence of autoantibodies. The large majority of the celiac disease patients express the HLA-DQ2.5 and/or HLA-DQ8 molecules [1], and gluten specific T-cells restricted to these HLA-molecules can be isolated from intestinal biopsies of the patients [2], [3]. The peptide binding motifs of HLA-DQ2.5 and HLA-DQ8 have been thoroughly investigated and both molecules show a preference for negative charges at certain anchor positions; DQ2.5 in positions P4, P6 and P7 [4]–[6] and DQ8 in positions P1 and P9 [7], [8]. Interestingly, these restricted T cells recognize gluten peptides only or preferentially after they undergo deamidation, a modification which is catalyzed by the enzyme transglutaminase 2 (TG2).

TG2 is a calcium-dependent enzyme which targets specific glutamine residues in peptides and proteins for either transamidation (crosslinking) or deamidation [9]. In the transamidation reaction, a stable isopeptide bond is formed between a peptidylglutamine residue (acyl-donor) and the amino group of an acyl-acceptor, e.g. peptidyllysine or a primary amine molecule. Deamidation of the targeted glutamine residue results in conversion to a glutamic acid and introduces a negative charge. It has been demonstrated that both for the deamidation [10] and transamidation reactions [11] the targeting of glutamine (Q) residues in peptides is strongly influenced by the positioning of C-terminally located proline (P) residues. Whereas a Q residue in the QXP consensus sequence is targeted by TG2, Q residues in a QP or QXXP sequence motif are not.

During the recent years several T-cell epitopes of wheat gluten have been identified [12]–[19]. The majority of these epitopes derives from the gliadin protein fraction of gluten and is presented to T cells in the context of HLA-DQ2.5. Notably, the gliadin epitopes often cluster in regions rich in proline residues. Conceivably, the selection of the T-cell epitopes is governed by several factors: (i) T-cell epitopes are usually spanning 9–12 residues and thus protection against complete digestion in the gastrointestinal tract imposed by proline residues is important [20]; (ii) another selective force is exerted by TG2 as these epitopes are dependent on deamidation by this enzyme; (iii) finally, epitope selection by HLA seems of importance as both HLA-DQ2.5 and HLA-DQ8 prefer binding of peptides with negatively charged anchor residues. Importantly, all these selective forces will act in concert.

Several recent studies have pointed towards a role of TG2 in the selection of T-cell epitopes in celiac disease. Using a set of synthetic overlapping peptides covering the whole sequence of a γ-gliadin protein, those peptides that were quickly deamidated by TG2 were also recognized by T-cell lines of celiac disease patients [11]. We have also demonstrated that the known HLA-DQ2.5-restricted gliadin epitopes are substrates for TG2, but the rate by which this modification occurs differs considerably between the peptides [21]. We found a correlation between the rate of deamidation of the different epitopes and their T cell immunostimulatory capacity. Finally, the finding that proline governs the specificity of the enzyme and that gluten T-cell epitopes are rich in proline residues further supports the notion of a selective force exerted by TG2.

In this study we aimed to shed further light on the role of TG2 in the selection of gluten T-cell epitopes. Gluten is an extreme complex mixture of diverse proteins. Several hundred distinct gluten proteins are belonging to the gliadin and glutenin fractions of a single wheat variety, and therefore tens of thousands different peptides will be present in a digest of wheat flour. To mimic the selection of T-cell epitopes from this vast sea of peptides that is present in the gut after gluten ingestion, we have developed a method to identify the preferred peptide substrates for TG2 in a proteolytic digest of whole gluten. Interestingly, we found that the great majority of preferred substrates for TG2 from this heterogeneous mixture are indeed peptides that harbor known gluten T-cell epitopes. We also discovered two peptides that harbor novel candidate T-cell epitopes.

## Materials and Methods

### Synthesis of peptides and purification of recombinant human TG2

Synthetic peptides were purchased from GL Biochem Ltd (Shanghai, China). Recombinant human TG2 was expressed in *Escherichia coli* with an N-terminal hexa-histidine tag and purified as described previously [22] with some minor modifications [23].

### Proteolysis of gluten

Whole gluten (Bob's Red Mill, Milwaukie, Oregon) digested with pepsin, trypsin, chymotrypsin, elastase and carboxypeptidase (PTCEC gluten) was a gift from M. Bethune (Stanford University) and was prepared as follows: A solution of 15 mg/ml gluten was dissolved in 0.01 N HCL (pH∼2.1) and was incubated with 0.6 mg/ml pepsin (American Laboratories) for 60 min at 37°C. The digest was adjusted to pH 6.5 with 50 mM sodium phosphate buffer and incubated with 0.375 mg/ml trypsin (Sigma), 0.375 mg/ml chymotrypsin (Sigma), 0.075 mg/ml elastase (Sigma) and 0.075 mg/ml carboxypeptidase A (Sigma) for 120 min at 37°C. The sample was heated to 95°C for 5 min to stop proteolysis followed by centrifugation and filtration of the supernatants.

### Validation of the enrichment procedure using synthetic peptides

To test the specificity of the enrichment procedure, 20 µM of the synthetic peptides DQ2-α-I (QLQPFPQPQLPY) and DQ2-γ-II (GIIQPQQPAQL) was separately mixed with 20 µM 5-biotinamido-pentylamine (5-BP) in 10 µl of 100 mM Tris/HCl pH 7.4 supplemented with 2 mM CaCl_2_. Both samples were incubated with 0.1 µg/µl TG2 at 37°C for 60 min before TG2 was inactivated by adding 1 µl iodoacetamide to a final concentration of 25 mM. A volume of 1 µl of each of the samples was added to 20 µl of 1 mg/ml PTCEC gluten digest. The sample was incubated with 200 µg Dynabeads M270 streptavidin (capacity: 200 pmol biotinylated peptides/1 mg beads) (Invitrogen Dynal AS, Norway) for 30 min in PBS/0.1% SDS followed by three washing steps each with 100 µl PBS/0.1% SDS and 100 µl 20% acetonitrile/water. For elution, beads were incubated with 15 µl 70% acetonitrile/2% formic acid/0.2 mM biotin for 30 min at 37°C and the eluate was analyzed by MALDI-TOF mass spectrometry (Ultraflex II, Bruker Daltonics, Bremen, Germany).

In order to rank the peptides according to their level of transamidation, a sample containing 20 µM of each of the peptide epitopes DQ2-α-II (PQPQLPYPQPQLPY), DQ2-γ-II (GIIQPQQPAQL), DQ2-γ-III (FPQQPQQPYPQQP) and DQ2-γ-IV (FSQPQQQFPQPQ) was incubated with 0.1 µg/µl TG2 and 20 µM 5-BP in 20 µl 100 mM Tris/HCl pH 7.4 with 2 mM CaCl_2_ at 37°C. Aliquots of 5 µl were removed after 1, 5 and 15 min incubation time and 1 µl iodoacetamide (final concentration 25 mM) was added to inactivate TG2. For enrichment, 1 µl of each of the samples was incubated with 150 µg Dynabeads M270 streptavidin for 30 min in PBS/0.1% SDS. Washing of the beads and elution of the enriched biotinylated peptides was performed as described above. MALDI-TOF spectra were acquired for the eluates of enriched transamidated peptides.

Further, 30 µM of a chymotryptic digest of α2-gliadin was incubated with 0.1 µg/µl TG2 in 25 µl 100 mM Tris/HCl pH 7.4 supplemented with 2 mM CaCl_2_ at 37°C for 60 min and the reaction was terminated by adding 1 µl iodoacetamide to a final concentration of 25 mM. The sample was purified by C_18_ ZipTips (Millipore, Billerica, MA, USA), the eluate was vacuum-dried, and the peptides were redissolved in 0.1% formic acid. Next, 300 fmol of the sample was analyzed by nano-LC coupled to a quadrupole-time-of-flight (Q-TOF) mass spectrometer (settings as described below). Similarly, 42 µM of a chymotryptic digest of α2-gliadin was incubated under identical conditions in the presence of 2 µM 5-BP. Transamidated peptides were enriched by incubating with 150 µg Dynabeads M270 streptavidin for 30 min in PBS/0.1% SDS followed by washing of the beads as described above. Enriched peptides were eluted by adding 10 µl of 70% acetonitrile/2% formic acid/0.2 mM biotin and incubating for 30 min at 37°C. Finally, MALDI-TOF spectra were acquired.

### Treatment of a PTCEC gluten digest with TG2 and enrichment of transamidated gluten peptides

A sample containing 1 mg/ml PTCEC gluten digest, 0.1 µg/µl TG2 and 200 pmol 5-BP in 100 mM Tris/HCl pH 7.4 supplemented with 2 mM CaCl_2_ (total volume of 20 µl) was incubated at 37°C for 30 min. After inactivation of TG2 by adding iodoacetamide (final concentration of 25 mM), the sample was incubated with 1 mg Dynabeads M270 streptavidin for 30 min in PBS/0.1% SDS and the beads were washed as described above. Enriched transamidated and biotinylated peptides were eluted from the beads by adding 25 µl of 70% acetonitrile/2% formic acid/0.2 mM biotin and incubating for 30 min at 37°C. Finally, the eluate was vacuum-dried.

### Analysis by nano-LC tandem mass spectrometry

The transamidated peptides enriched from PTCEC gluten were analyzed either by a Q-TOF hybrid mass spectrometer (MicroTof-q, Bruker Daltonics, Bremen, Germany) or an Orbitrap mass spectrometer (LTQ Orbitrap XL, Thermo Scientific, Bremen, Germany), both coupled to a nano-LC system. For nano-LC-Q-TOF analysis, dried samples were dissolved in 8 µl 0.1% trifluoroacetic acid and a volume of 6 µl was injected into an Agilent 1100 series nano-LC system (Agilent Technologies, Palo Alto, CA). Peptides were separated on an analytical column (150 mm×0.075 mm) packed with 100 Å C18 3.5 µm particles (G&T Septech AS, Norway). A linear gradient of 2–60% solvent B in 60 min was applied with a flow rate of 300 nL/min (solvent A: 0.1% acetic acid/water, solvent B: 0.1% acetic acid/acetonitrile). The Q-TOF was operated in the data-dependent mode to automatically switch between MS and MS/MS acquisition. Collision energies were set between 30–50 eV depending on the charge state of the precursor ions. Data were acquired using microTOFControl v2.0 and processed using DataAnalysis v3.4. For nano-LC-Orbitrap analysis, the peptides were purified by C_18_ ZipTips (Millipore, Billerica, MA, USA), vacuum-dried and redissolved in 10 µl of 1% formic acid, and then 3 µl were injected into an Ultimate 3000 nanoLC system (Dionex, Sunnyvale, CA, USA). For separation of peptides, an Acclaim PepMap 100 column (120 mm×0.075 mm) packed with 100 Å C18 3 µm particles (Dionex) was used. A flow rate of 300 nL/min was employed with a solvent gradient of 7–35% B in 77 min and then from 35% to 50% B in 10 min (solvent A: 0.1% formic acid/water; solvent B: 0.1% formic acid/90% acetonitrile). The mass spectrometer was operated in the data-dependent mode to automatically switch between Orbitrap-MS and LTQ-MS/MS acquisition. Data were acquired using Xcalibur v2.5.

### Identification of enriched transamidated gluten peptides

First, a database consisting of all known protein sequences derived from *Triticum aestivum* was built by extracting entries (n = 4654) from the UniprotKB database (release 15.0) at the European Bioinformatics Institute (EBI) using the SRS server. The LC-MS/MS data were searched against this database using the Mascot search engine and Proteome Discoverer software version 1.0 (Thermo Fisher Scientific Inc., Waltham, MA). In Mascot the glutamine modification by 5-BP linkage (mass increase of 311.17 Da) was implemented and selected as variable modification together with deamidation of glutamine and aspargine and pyro-glutamate formation. For analysis of LTQ-Orbitrap data, the mass tolerance was set as 10 ppm for the precursor ion and 0.6 Da for the fragment ions, whereas for analysis of Q-TOF data the mass tolerance was set as 0.5 Da for the precursor ion and 0.2 Da for the fragments ions. All reported hits were in addition manually interpreted. If the glutamine residues targeted by TG2 could not be unambiguously assigned based on the MS/MS spectrum, also the established rules governing the targeting of glutamine residues by TG2 were considered [10], [11]. In a few cases, extracted ion chromatograms were generated to distinguish peptides that carry both transamidated and deamidated glutamine residues from peptides harboring only a transamidated glutamine residue.

### T-cell assay

Twenty polyclonal T-cell lines derived from intestinal biopsies of celiac disease patients as previously described [16] were tested for recognition of the gluten peptides. Synthetic peptides representing peptides #9 (VPVPQLQPQNPSQQQPQEQVPL), #19 (PHQPQQQVPQPQQPQQPF), #25 (SHQQQPFPQQPYPQQPYPS), and #30 (SFPQPQPQQPQQPS) were tested in T-cell proliferation assays after TG2 treatment done by incubating 100 µM peptide with 0.1 µg/µl TG2 in 100 mM Tris/HCl pH 7.4 buffer supplemented with 2 mM CaCl_2_ for 120 min at 37°C. A panel of synthetic peptides representing already known T-cell epitopes was included in the same assay as reference. Sixty thousand HLA-DQ2 homozygous EBV-transformed B lymphoblastoid cells were irradiated (75 Gy) and incubated overnight with different concentrations of the TG2-treated peptides. On day one, 40 000 T cells were added. On day three, 1 µCi ^3^H-thymidine was added per well and T-cell proliferation was measured on day 4 as thymidine incorporation. The HLA-restriction of the T-cell lines TCL.422.02.2.4 and TCL.497.C.1.3 was assessed by blocking recognition of TG2-treated peptide #25 (10 µM) using 5 µg/ml anti-DR (B8.11), anti-DQ (SPV-L3), or anti-DP (B7/21) specific monoclonal antibodies during B cell and antigen co-incubation.

## Results

### Validation of the procedure to identify the best TG2 substrates

**Figure 1:**
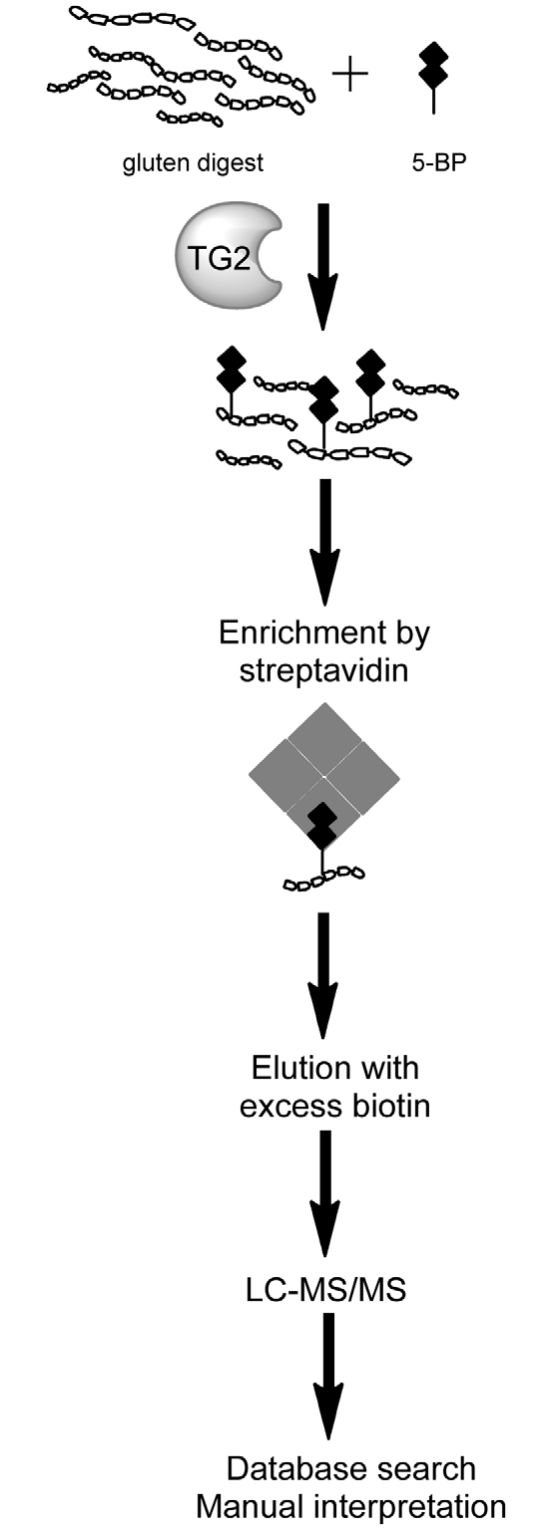
Schematic view of the established method to enrich and analyze TG2 peptide substrates. A PTCEC digest of wheat gluten was mixed with a small amount of 5-BP which served as a substrate for TG2 in a transamidation reaction. The transamidated, biotinylated peptides were enriched from the digest using magnetic streptavidin beads, eluted with an excess of biotin and analyzed by LC-MS/MS. Database searching was performed using a database made up of all entries of *Triticum aestivum* present in the Uniprot database. In addition, MS/MS spectra were manually inspected.

**Figure 2:**
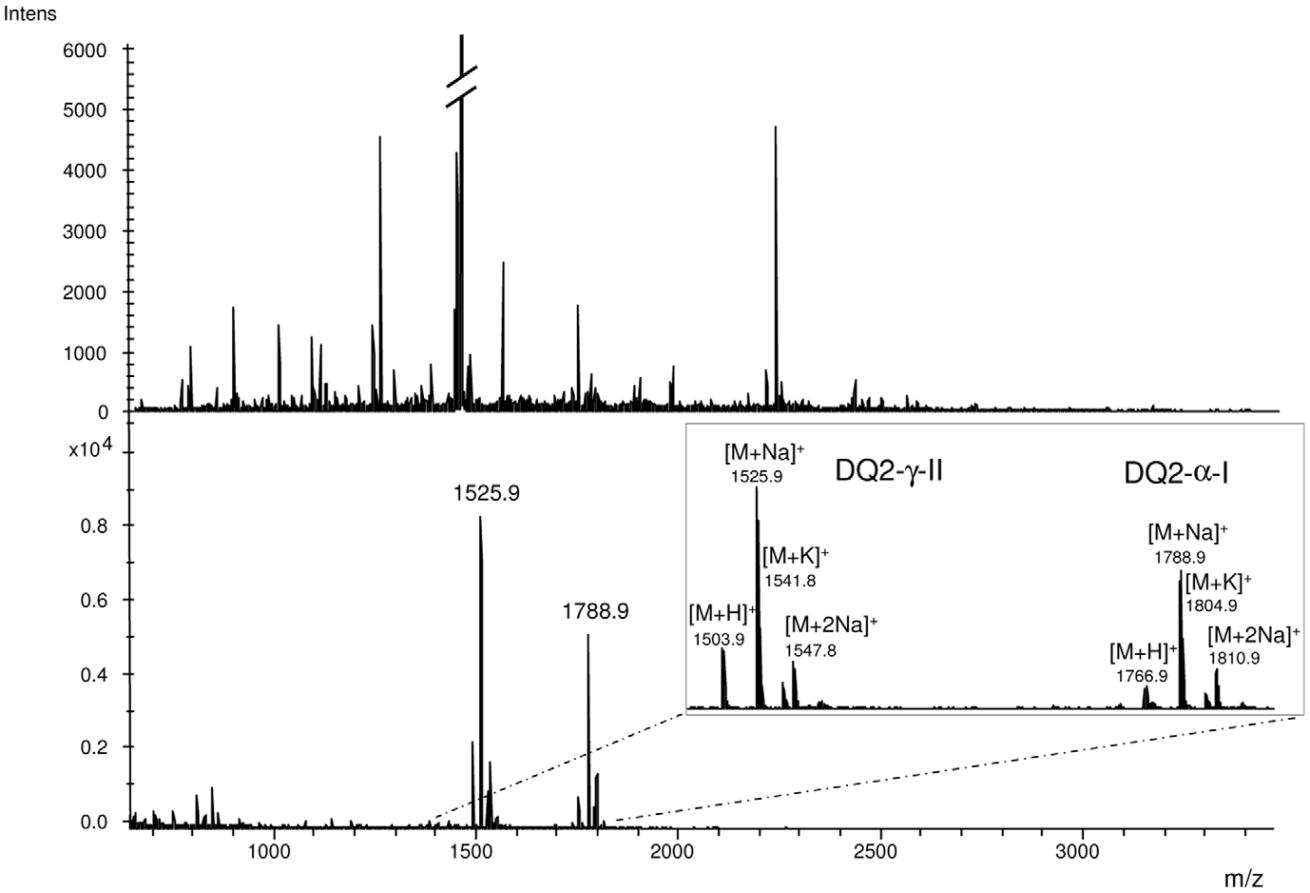
Specific enrichment of transamidated peptides. A PTCEC digest of whole gluten was spiked with the transamidated DQ2-α-I and DQ2-γ-II peptides. MALDI-TOF spectra before (upper panel) and after enrichment (lower panel) are shown. The inset shows the four signals obtained for each of the two enriched peptides that correspond to the transamidated peptide, its sodium adduct, its potassium adduct and the adduct with two sodium ions, respectively. All signals observed in the lower mass range (up to m/z 900) were derived from matrix clusters.

Incubation of a PTCEC digest of whole wheat gluten with small amounts of the primary amine 5-BP and TG2 will result in transamidation of a limited number of gluten peptides. In order to detect and identify these TG2 peptide substrates in such a heterogeneous peptide mixture, transamidated and hence biotinylated peptides were enriched using magnetic streptavidin beads (Fig. 1). First, we tested the specificity of the enrichment method for transamidated peptides. Two synthetic peptides known to be substrates for TG2, DQ2-α-I and DQ2-γ-II, were each incubated with TG2 in the presence of 5-BP. Both samples were spiked into a PTCEC digest of gluten and streptavidin beads were added. Analysis by MALDI-TOF mass spectrometry before and after enrichment demonstrated a high specificity of the enrichment procedure as only the transamidated DQ2-α-I and DQ2-γ-II peptides were detected after elution from streptavidin beads (Fig. 2). The transamidated peptides were not observed prior to enrichment.

We next tested whether the deamidation and transamidation reactions catalyzed by TG2 prefer the same gluten peptides. Previously we have shown that epitope peptides DQ2-α-II and DQ2-γ-II undergo rapid deamidation by TG2, whereas epitope peptides DQ2-γ-III and DQ2-γ-IV are poorly deamidated [21]. Equal amounts of these four peptides were mixed with a limited amount of 5-BP and incubated with TG2 for different periods of time. The transamidated peptides were enriched from the samples and MALDI-TOF spectra were acquired. The relative signal intensities of the transamidated DQ2-α-II and DQ2-γ-II epitope peptides increased much faster over time than those of the DQ2-γ-III and DQ2-γ-IV epitope peptides suggesting that the former are better substrates of TG2 (data not shown). This finding is consistent with previously reported data for deamidation of these four peptides [21]. Further, we digested a single recombinant α2-gliadin protein by chymotrypsin and we used this more heterogeneous gluten peptide mixture to test which peptides were deamidated and transamidated. For both reactions, the α33mer peptide (Table 1) and the peptide VRVPVPQLQPQNPSQQQPQEQVPL were identified as substrates. Thus we concluded that enrichment of gluten peptides by means of crosslinking with 5-BP can be used to provide information as to which peptides in a heterogeneous mixture are preferred also for the deamidation reaction. The method can also determine which glutamine residues within the preferred substrates are targeted by the enzyme.

**Table 1:** Identified T-cell epitopes in wheat gluten and their observed T-cell response in celiac disease patients.

T-cell epitopes	Peptide binding register (P1–P9)	T-cell response	Identified
DQ2-α-I	P F P Q P **Q** L P Y	8/14 [34] 12/17 [12] 7/20 [17]	√
DQ2-α-II	P Q P **Q** L P Y P Q	14/14 [34] a 10/20 [17] a 11/17 [12] a	√
DQ2-α-III	P Y P Q P **Q** L P Y	14/14 [34] a 10/20 [17] a 11/17 [12] a	√
DQ2-γ-I	P Q Q S F P **Q** Q **Q**	3/14 [34]	
DQ2-γ-II	I Q P **Q** Q P A Q L	4/14 [34] 5/13 [14]	√♦
DQ2-γ-III/DQ8-γ-I	**Q** Q P **Q** Q P Y P **Q**	6/14 [34] b	
DQ2-γ-IV	S Q P **Q** Q **Q** F P Q	3/13 [14]	
DQ2-γ-VI	**Q** Q P F P **Q** Q P **Q**	6/14 [34] b	
DQ2-γ-VII	P Q P **Q** Q **Q** F P Q	-	
DQ2-γ-VIIb/DQ8-γ-I	**Q** Q P **Q** Q P F P **Q**	3/13 [14]	√
Glia-α20	F R P **Q** Q P Y P Q	4/20 [17]	√♦
Glt-17	P F S **Q** Q **Q** Q P V	c	√
DQ8-α-I	**Q** G S F Q P S Q **Q**	-	
DQ8-glutenin	**Q** G Y Y P T S P **Q**	-	
ω17mer	QP**Q**QPFPQPEQPFPWQP	5/14 [30]	
γ26mer	FLQP**Q**QPFP**Q**QP**Q**QPYP**Q**QP**Q**QPFPQ	-	
α33mer	LQLQPFPQP**Q**LPYPQP**Q**LPYPQP**Q**LPYPQPQPF	14/14 [34] 13/13 [14]	

Glutamine residues expected to be targeted by TG2 are given in bold.

a Peptide PQPQLPYPQPQLPY harboring both epitopes, DQ2-α-II and DQ2-α-III, was tested.

b Peptide LQPQQPFPQQPQQPYPQQPQ harboring both epitopes, DQ2-γ-III/DQ8-γ-I and DQ2-γ-VI, was tested.

c Note that this peptide is not identical to peptide QQPPFSQQQQQPLPQ which was previously known as the “Glt-17” epitope. Peptide QQPPFSQQQQQPLPQ has previously been tested for T-cell response in CD patients: 3/20 [17].

♦Identified TG2 peptide substrates that harbor incomplete 9mer core binding regions.

### Identification of enriched gluten peptides by mass spectrometry

In order to identify optimal TG2 gluten peptide substrates, a PTCEC digest of whole gluten was incubated with TG2 in the presence of limiting amounts of 5-BP for 30 minutes. After specific enrichment of transamidated gluten peptides by magnetic streptavidin beads, eluted peptides were identified by LC-MS/MS analysis and subsequent database searching (Fig. 1). Altogether, 31 different peptide sequences were identified from the enriched sample of transamidated gluten peptides (Table 2). The targeted glutamine (Q) residues in these peptides were typically in the QXP-motif which is in accordance with previous results regarding TG2 specificity [10], [11]. Remarkably, the majority of the identified peptides contained complete or truncated versions of known gluten T-cell epitopes (Tables 1 and 2). Among the identified nine α-gliadin derived peptides, five peptides harbored at least two of the DQ2-α-I, DQ2-α-II or DQ2-α-III epitopes. The DQ2-α-II epitope was present in all of the five peptides in one or two copies (peptides #1–5), while the DQ2-α-I and DQ2-α-III epitopes were present in peptides #3–5 and peptides #1–3, respectively. The α-gliadin derived peptide #6 harbored an incomplete Glia-α20 epitope. The α-gliadin derived 33mer peptide that is known to be a superior TG2 substrate was not identified. However, several isoforms (peptides #3 and #5) and truncated versions (peptides #1, #2, and #4) of this peptide were observed. The α-gliadin derived peptides #7–9 did not harbor any known T-cell epitopes. Peptide #7 is similar to the p31–44 “toxic” peptide previously reported except a L to P substitution at the N-terminus [24].

**Table 2:** TG2 peptide substrates identified by nano-LC MS/MS.

#	Peptide identified	Modifications	Protein (a)	T-cell epitope	Intact 9mer core	
**1**	PQPQLPYPQP**Q**LPYPQPQPF	5-BP: Q11	α-gliadin (3)	DQ2-α-II (2×), DQ2-α-III		Yes
	PQP**Q**LPYPQPQLPYPQPQPF	5-BP: Q4				Yes
	PQP**Q**LPYPQP**Q**LPYPQPQPF	5-BP: Q11, DA. Q4				Yes
**2**	LPYPQP**Q**LPYPQPQPF	5-BP: Q7	α-gliadin (3)	DQ2-α-II, DQ2-α-III		Yes
**3**	QLQPFPQP***Q***LPYPQP***Q***LPYPQPQPF	5-BP: Q9/Q16^b^	α-gliadin (3)	DQ2-α-I, DQ2-α-II (2×), DQ2-α-III	Yes	
	QLQPFPQP***Q***LPYPQP***Q***LPYPQPQPF	5-BP: Q9/Q16, DA: Q9/Q16^c^			Yes	
**4**	QLQPFPQP**Q**LPYPQPQ	5-BP: Q9	α-gliadin (5)	DQ2-α-I, DQ2-α-II		Yes
**5**	QLQPFPQP**Q**LPYPQPQPF	5-BP: Q9	α-gliadin (4)	DQ2-α-I, DQ2-α-II		Yes
**6**	RP**Q**QPYPQPQPQ	5-BP: Q3	α-gliadin (9)	Glia -α20		No
**7**	LG***QQ***QPFPPQQPYPQPQPFPS	5-BP: Q3/Q4^b^	α-gliadin (3)			
**8**	LQPQNPSQQQPQE**Q**VPL	5-BP: Q14	α-gliadin (6)			
	LQPQNPSQ**Q**QPQE**Q**VPL	5-BP: Q14, DA: Q9				
**9**	VPVPQLQPQNPSQQQPQE**Q**VPL	5-BP: Q19	α-gliadin (4)			
	VPVPQLQPQNPSQ**Q**QPQE**Q**VPL	5-BP: Q19, DA: Q14				
**10**	GIIQP**Q**QPA	5-BP: Q6	γ-gliadin (20)	DQ2-γ-II		No
**11**	VQG**Q**GIIQPQ	5-BP: Q4^d^	γ-gliadin (20)	DQ2-γ-II		No
**12**	VQG**Q**GIIQPQQ	5-BP: Q4	γ-gliadin (20)	DQ2-γ-II		No
**13**	VQG**Q**GIIQPQQPA	5-BP: Q4	γ-gliadin (20)	DQ2-γ-II		No
	VQG**Q**GIIQP**Q**QPA	5-BP: Q4, DA: Q10				No
**14**	VQG**Q**GIIQPQQPAQ	5-BP: Q4	γ-gliadin (20)	DQ2-γ-II		No
**15**	LVQG**Q**GIIQPQQPA	5-BP: Q5	γ-gliadin (20)	DQ2-γ-II		No
**16**	LVQG**Q**GIIQPQQPAQ	5-BP: Q5	γ-gliadin (20)	DQ2-γ-II		No
**17**	QLVQG**Q**GIIQPQQPAQ	5-BP: Q6	γ-gliadin (20)	DQ2-γ-II		No
**18**	S**Q** QPQQPFPQPQ or S**Q** QPQQPFPQQP	5-BP: Q2	γ-gliadin (9)	DQ2-γ-VIIb/DQ8-γ-I		Yes
**19**	PHQPQQQVPQP**Q**QPQQPF	5-BP: Q12	γ-gliadin (7)	New candidate epitope		Yes
**20**	S***QQQ***QPVLPQQQPV e	5-BP: Q2/Q3/Q4^b^	LMW glutenin (2)	Glt-17		No
**21**	SQQQPPFSQQ**Q**QPV	5-BP: Q11	LMW glutenin (6)	Glt-17		Yes
**22**	SQQQPPFS***QQQ***QPVxPQQPS e	5-BP: Q9/Q10/Q11^b^	LMW glutenin (2/3)	Glt-17		Yes
**23**	S***QQQ***QPVxPQQPS e	5-BP: Q2/Q3/Q4^b^	LMW glutenin (2/1)	Glt-17		No
**24**	PQQPPFSQQ**Q**QPVLPPQQSPFPQ e	5-BP: Q10	LMW glutenin (5)	Glt-17		Yes
**25**	SHQQQPFPQQPYP**Q**QPYPS f	5-BP: Q14	LMW glutenin (1)	New candidate epitope		Yes
	SHQQQPFP**Q**QPYPQQPYPS f	5-BP: Q9				Yes
**26**	PQQPPFSQ***QQ***QPx	5-BP: Q9/Q10^b^	LMW glutenin (3)	Cross-reactive to Glt-17		Yes
**27**	SQQQQPPFSQQQPPFSQQQ**Q**QPL	5-BP: Q20	LMW glutenin (1)	Cross-reactive to Glt-17		Yes
**28**	QQQPPFSQ**Q**QPx	5-BP: Q9	LMW glutenin (11)			
**29**	S**Q**QQLPPFSQQQSPF	5-BP: Q2	LMW glutenin (1)			
**30**	SFPQPQP**Q**QPQQPS	5-BP: Q8	LMW glutenin (1), ω-gliadin (1)			
**31**	S***QQQ***QLFPQQPS	5-BP: Q2/Q3/Q4^b^	LMW glutenin (1)			

Glutamine residues targeted by TG2 are given in bold.

DA, deamidation

The 9mer core region of T-cell epitopes are underlined.

“x” indicates I or L.

a number of protein entries in database.

b not possible to determine which Q residue is transamidated (shown in bold and italic).

c not possible to determine which Q residue is deamidated and which is transamidated.

d not possible to determine whether Q2 or Q4 is targeted; however, this sequence has previously been shown to be targeted at Q4 [17]. The same Q residue is expected to be targeted in peptides #12–17.

e identified in a sample incubated with TG2 for one minute.

f two possible 9mer binding registers to HLA-DQ2.5.

Ten peptides derived from γ-gliadin were identified, and eight of these peptides contained parts of the DQ2-γ-II epitope (peptides #10–17). Notably, these peptides shared the same peptide core region but differed in their N- and C-terminal extensions. The DQ2-γ-VIIb/DQ8-γ-I epitope was present in one of the identified γ-derived peptides (peptide #18).

Twelve identified peptides derived from glutenin proteins. The complete DQ2-restricted glutenin-17 epitope, Glt-17, was present in three of these peptides (peptides #21, #22 and #24) while two of the peptides (peptides #20 and #23) harbored truncated versions of the epitope. Interestingly, several of the other identified TG2 peptide substrates of glutenin proteins have sequences similar to known epitopes.

### Identification of two novel candidate T-cell epitopes

**Figure 3:**
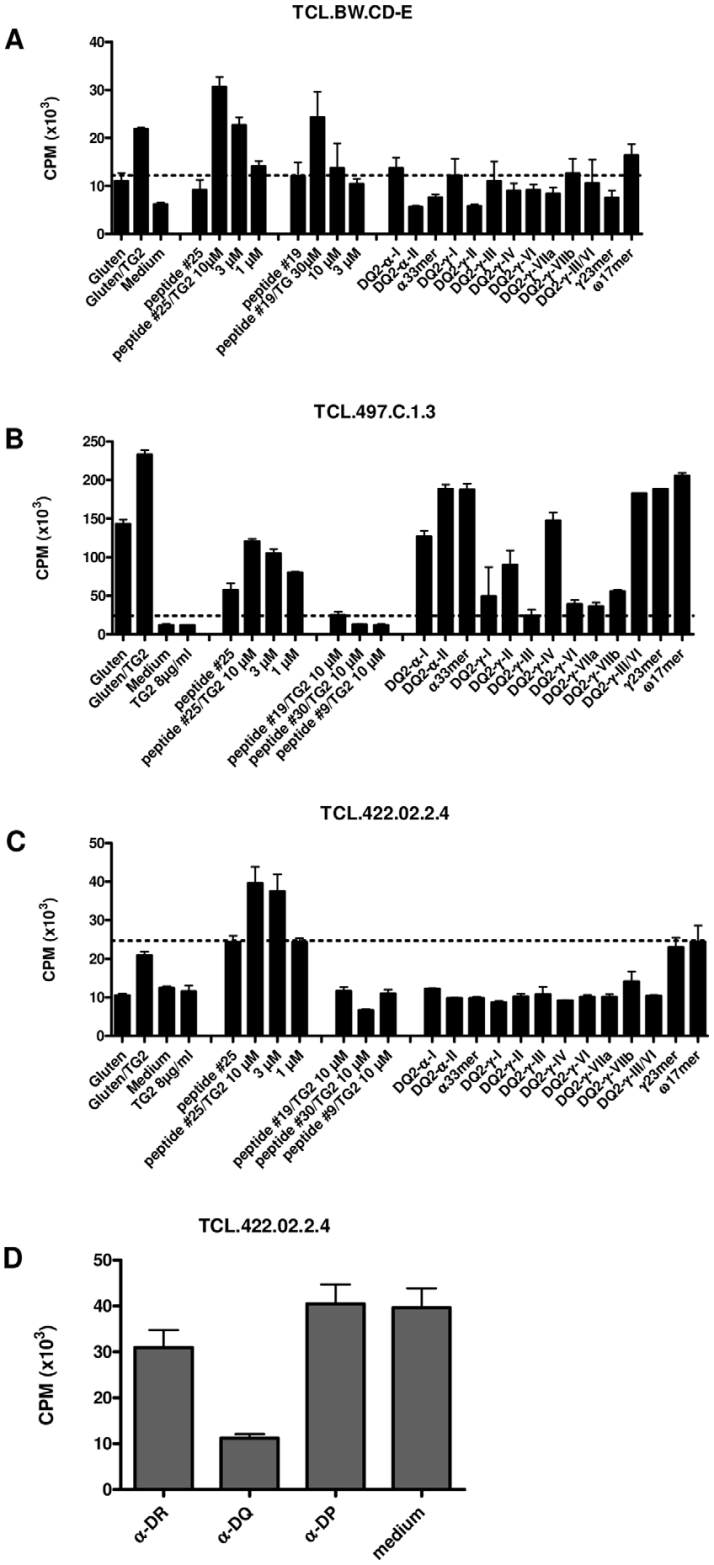
T-cell recognition of gluten peptides #19 and #25. The identified TG2 peptide substrates VPVPQLQPQNPSQQQPQEQVPL (peptide #9), PHQPQQQVPQPQQPQQPF (peptide #19), SHQQQPFPQQPYPQQPYPS (peptide #25) and SFPQPQPQQPQQPS (peptide #30) were tested together with a panel of synthetic epitope peptides for recognition by three T-cell lines, TCL.BW.CD-E (A), TCL.497.C.1.3 (B), and TCL.422.02.2.4 (C), generated from biopsies of HLA-DQ2.5-positive celiac disease patients. The sequences of the other synthetic peptides tested were|: DQ2-α-I, QLQPFPQPELPY; DQ2-α-II, PQPELPYPQPQLPY; α33mer, LQLQPFPQPELPYPQPELPYPQPELPYPQPQPF; DQ2-γ-I, pyroglutamic acid-PQQPQQSFPEQQRP (in A), pyroglutamic acid-PEQPQQSFPEQERP (in B and C); DQ2-γ-II, GIIQPEQPAQL; DQ2-γ-III, FPEQPEQPYPEQ (in A), FPQQPEQPYPQQ (in B and C); DQ2-γ-IV, FSQPEQEFPQPQ; DQ2-γ-VI, PEQPFPEQPEQ; DQ2-γ-VIIa, TEQPEQPFPQP; DQ2-γ-VIIb, FPQPEQEFPQPQ; DQ2-γ-III/DQ2-γ-VI, LQPEQPFPEQPEQPYPEQPQ; γ23mer, EQPFPEQPEQPYPEQPEQPFPQP; ω17mer, QPQQPFPQPEQPFPWQP. Note that peptides #25 and #19 were tested at different concentrations. The α33mer peptide was tested at 2 µM while the other peptides were tested at 10 µM. Responses were measured in a proliferation assay by the incorporation of ^3^H-thymidine (counts per minute (CPM)×10^3^). The dashed line indicates two-fold background proliferation observed with medium only. Peptides #25 and #19 were tested in triplicates; other antigens were tested in duplicates. Error bars indicate the standard error of mean. The experiment was repeated twice. The HLA-restriction of the T-cell line TCL.422.02.2.4 was assessed by blocking recognition of peptide #25 using anti-DR, anti-DQ and anti-DP specific monoclonal antibodies (D). Similar results were obtained for T-cell line TCL.497.C.1.3.

Five of the identified TG2 peptide substrates that did not contain any known T-cell epitopes had sequences suggesting potential binding to HLA-DQ2.5 (a negative charge in one of the anchor positions P4, P6 or P7 [4]–[6]; no proline residues in positions P2, P4, P7 or P9 [14]). In an attempt to identify new T-cell epitopes, we therefore tested these peptides for recognition by twenty gluten reactive, polyclonal T-cell lines generated from intestinal biopsies of HLA-DQ2.5 positive celiac disease patients. The following peptides were tested: peptide #9 (VPVPQLQPQNPS*Q****Q****QPQE****Q*** *VP*L) which also contains peptide #8, peptide #19 (PHQPQQQVPQP**Q**QPQQPF), peptide #25 (SHQQQPFP***Q****QPY* *P* ***Q*** *QPY*PS) and peptide #30 (SFPQPQP**Q**QPQQPS; predicted HLA-DQ2.5 binding registers are underlined or in italics; Q residues targeted by TG2 are in bold). Peptides were treated with TG2 and tested together with a panel of known gluten epitopes as reference in a T-cell proliferation assay. While none of the T-cell lines responded to peptides #9 and #30, the three T-cell lines TCL.BW.CD-E, TCL.497.C.1.3 and TCL.422.02.2.4 responded to peptide #25 in a TG2-dependent and dose-dependent fashion (Fig. 3A–C). Interestingly, peptide #25 was the only peptide which T-cell line TCL.422.02.2.4 responded to, indicating that there is no cross-reactivity between this peptide and the DQ2-γ-VI epitope which has a very similar sequence. The T-cell line TCL.BW.CD-E in addition gave a weaker, but specific response to peptide #19. We thus conclude that peptide #25 and peptide #19 harbor two novel candidate T-cell epitopes.

### Transamidation and deamidation of distinct glutamine residues within the same peptide

Four α-gliadin and one γ-gliadin derived TG2 peptide substrates were observed as derivatives carrying both a transamidated and a deamidated glutamine residue. For peptide #1 (PQPQLPYPQPQLPYPQPQPF), transamidation and deamidation sites could be assigned to Q11 and Q4 (underlined), respectively. A transamidated and deamidated derivative was also observed for an elongated version of peptide #1. In this 25mer α-gliadin peptide (peptide #3, QLQPFPQPQLPYPQPQLPYPQPQPF, targeted Q residues underlined), it could however not be unambiguously determined which of the two targeted glutamine residues (Q9 and Q16) was transamidated or deamidated. For peptide #8 (LQPQNPSQQQPQEQVPL), Q14 was found to be transamidated whereas Q9 was deamidated. These modifications were observed for the same glutamine residues in peptide #9 which represents an N-terminally extended derivative of peptide #8. The γ-gliadin derived peptide VQGQGIIQPQQPA (peptide #13) was identified to be transamidated at the Q4 residue and deamidated at the Q10 residue.

## Discussion

In this study we have identified the preferred substrates of TG2 in proteolytic digests of whole wheat gluten. To simplify the identification we established an enrichment method which is based on the TG2-mediated transamidation reaction followed by sequence determination by mass spectrometry. We identified 31 different peptide substrates of TG2. Strikingly, the majority of these peptides harbor known gluten T-cell epitopes.

To date, more than 15 HLA-DQ2.5 and HLA-DQ8-restricted gluten derived T-cell epitopes have been identified [12]–[19]. For the majority of HLA-DQ2.5-restricted gliadin epitopes, the kinetics of TG2-mediated deamidation was recently determined and large variations between the peptide epitopes were observed [21]. Interestingly, there seems to be a correlation between the level of deamidation of the different epitopes and how frequently the epitopes are recognized by T-cells of celiac disease patients.

Peptide substrates of TG2 can be either transamidated or deamidated. Deamidated reaction products generated in the absence of primary amines can be directly detected by LC-MS in mixtures of low complexity, e.g. in a chymotryptic digest of a single recombinant gliadin protein. However, in our hands this approach was not feasible for the identification of the best substrates of TG2 when analyzing the PTCEC digest of whole gluten because of its extreme heterogeneity. Although the propensity for either of the two modifications may differ slightly for some peptide substrates, the substrate specificity for deamidation and transamidation overall appears very similar [23]. We confirmed this notion, as we found the same two peptides being substrates in the deamidation and transamidation reaction when testing a chymotryptic digest of a recombinant gliadin protein. Moreover, when testing substrate potency of four peptides for transamidation and deamidation, we observed the same rank order for the peptides in both reactions. We therefore decided to harness the transamidation reaction to identify the best peptide substrates in the complex peptide mixture of a PTCEC digest of whole gluten. By this approach, we were able to tremendously reduce the sample complexity by enriching for the TG2-modified peptides and thereby facilitating identification of individual peptides.

The transamidation reaction in this highly competitive environment was performed in the presence of limited amounts of 5-BP and short incubation periods. It is striking that the majority of the identified transamidated peptides contained intact known T-cell epitopes or truncated versions thereof. Five of the nine identified α-gliadin derived peptides carry more than one copy of either of the epitopes DQ2-α-I, DQ2-α-II, or DQ2-α-III, and eight of the ten identified γ-gliadin peptides harbor a part of the DQ2-γ-II epitope. These epitopes showed also the highest deamidation rates in our recent study [21], and they constituted nearly half of the gluten-TG2 substrates we identified.

The identified TG2 substrates that carry T-cell epitopes can be dichotomized according to whether they harbor complete or incomplete 9mer core binding regions. Interestingly, this classification correlates with the frequency by which the T-cell epitopes are recognized by T cells of celiac disease patients. T-cell epitopes that are frequently recognized by T cells of celiac disease patients (e.g. DQ2-α-I, DQ2-α-II/DQ2-α-III) were represented by their intact 9mer sequences whereas the T-cell epitopes that are infrequently recognized (e.g. DQ2-γ-II, Glia-α20, Glt-17) were represented by partial 9mer sequences. This was particularly striking for the DQ2-γ-II epitope which was never represented by a complete 9mer, but appeared in C-terminally truncated forms in 8 distinct peptides. In keeping with our results, Shan *et al.* reported that the DQ2-γ-II epitope was not among the most proteolytically stable fragments of a recombinant γ-gliadin protein [20]. These data underscore that proteolytic degradation is an important force in the selection of gluten T-cell epitopes in celiac disease.

Among the peptides selected as substrates by TG2 we identified sequences that are likely to contain novel T-cell epitopes. Five peptides that did not harbor already known T-cell epitopes were suspected to be T-cell stimulatory as their sequences indicated potential HLA-DQ2.5 binding. We tested synthetic peptides representing these five sequences for recognition by twenty polyclonal T-cell lines of HLA-DQ2.5-positive celiac disease patients. Two of them, peptide #19 and peptide #25, elicited dose-dependent T-cell responses in one or more T-cell lines. Peptides #8 and #9, which are found in the well-investigated α2-gliadin protein, have also been reported as substrates for TG2 [25] and their sequences suggest that they may bind to HLA-DQ2.5. In keeping with previous findings [12], [26], our results indicate however that these peptides do not harbor T-cell epitopes. The sequences of the remaining peptides (#7, #28, #29, and #31) do not contain obvious binding motifs of HLA-DQ2.5 or HLA-DQ8, and they are thus unlikely to be efficient stimulators of T cells from celiac disease patients.

The immunostimulatory α-gliadin derived 33mer fragment, which is a very good TG2 substrate and which is resistant to proteolysis [27], was not among the identified TG2 gluten substrates. Recently, the occurrence of T-cell epitopes in α-gliadin proteins of different cultivars was reported [28]. This study found, as was indicated in an earlier study [29], that the complete α33mer fragment only exists in α-gliadin proteins encoded in the D-genome of *Triticum aestivum*. Thus, the α33mer peptide was likely present in a low amount in the PTCEC gluten digest we used which could explain its absence among the identified TG2 substrates. We observed, however, several variants of the α33mer peptide (peptides #3 and #5) and as well as truncated versions of these peptides (peptides #1, #2, and #4). Notably, it has been demonstrated that such shorter versions can be as potent as the α33mer peptide in stimulating intestinal T-cell responses [30].

Among the identified TG2 peptide substrates a few peptides were observed as transamidated and deamidated at the same time. These findings may have implications for the generation of TG2-specific autoantibodies in celiac disease whose appearance is strictly dependent on the dietary intake of gluten [31]. In a previously proposed hapten-carrier model, uptake of covalent complexes between TG2 and gluten peptides by TG2-specific B cells was suggested to result in activation of gluten-specific T cells which subsequently provide help for the B cells to secrete antibodies [32]. In accordance with this hypothesis, cross-linking of gluten peptides to TG2 via generation of isopeptide bonds between glutamine residues in the peptide and lysine residues of TG2 has been described [33]. If those TG2-bound gluten peptides carry an additional deamidated glutamine residue in a T-cell epitope distant from the transamidation site, e.g. as observed for peptides #1 and #3, presentation of this epitope would only require cleavage of the peptide backbone by proteases present in the endocytic compartments while leaving the isopeptide bond intact.

In a heterogeneous gluten digest, TG2 is not targeting all the different gluten peptides, but shows a clear preference for those which are found as T-cell epitopes in celiac disease patients. This finding argues for TG2 exerting a strong pressure in the selection of T-cell epitopes in celiac disease. Our data also demonstrate the important role of proteolysis in the selection of T-cell epitopes as the T-cell epitopes that are frequently recognized by T cells of celiac disease patients more often remain intact than the epitopes that are infrequently recognized. Together, the selective forces exerted by TG2 and gastrointestinal proteolysis are key factors to determine the repertoire of gluten epitopes in celiac disease.
